# Measuring the attractiveness of trip destinations based on human mobility data

**DOI:** 10.1038/s41598-025-29023-0

**Published:** 2025-11-27

**Authors:** Keisuke Kondo

**Affiliations:** 1 Research Institute of Economy, Trade and Industry, Chiyoda, Tokyo, 100-8901 Japan; 2 Research Institute for Economics and Business Administration, Kobe University, Kobe, Hyogo 657-8501 Japan

**Keywords:** Regional attractiveness index, Person trip survey, Human mobility data, Gravity equation, Exploratory spatial data analysis, Applied mathematics, Computational science, Software, Statistics

## Abstract

This study proposes a novel concept of a regional attractiveness index based on human mobility flows. Assuming that individuals’ mobility choice is based on utility maximization, this study aims to recover the attractiveness of trip destinations by estimating the gravity equation for interregional trip flows. Using mobility data from a person trip survey and mobile phone data in Japan, this study investigates whether different trip purposes (e.g., commuting to office and school, recreational trips, business trips, and returning home) and seasonal and tourism factors (e.g., holidays, events, and amusement facilities) can reveal spatial and temporal variations in the attractiveness of trip destinations. This study found that the proposed approach using interregional trip flows can effectively capture the extent to which trip destinations attract people. As real-time human mobility data become increasingly available, the new index of regional attractiveness is expected to become a key performance indicator for daily monitoring of urban and regional economies.

**Supplementary Information:**

The online version contains supplementary material available at 10.1038/s41598-025-29023-0.

## Introduction

In a manner analogous to the assessment of vital signs that inform healthcare decisions, it is imperative to establish indicators that serve as vital signs for comprehending the status of the regions. While numerous potential indicators exist, the present study endeavors to measure regional attractiveness as one of these vital signs from a neutral perspective, independent of population size or economic activity scale.

The dimensions of regional attractiveness are characterized by their diversity, subjectivity, and qualitative nature^1^. For instance, some regions are quiet on weekdays but crowded on weekends and holidays, and regional attractiveness can change with the seasons. Furthermore, the attractiveness of a region can undergo fluctuations with the change in seasons. Moreover, when assessing regional attractiveness as a vital sign, it is imperative that the underlying data is stable and continuously observed. While questionnaires are a viable method for assessing regional attractiveness, they are often time-consuming and costly. Furthermore, the information obtained through this method is not immediately available when it is needed.

In recent years, technological advancements and the increased availability of big data have made human mobility data a potential source for measuring a region’s vital signs. Given data that traces human mobility, we must ask why people choose a particular region as their travel destination over many other possible regions. This study quantitatively evaluates regional attractiveness by constructing a theoretical model of people’s choice of trip destination and combining it with human mobility data. The evaluation is based on the preferences that led people to visit a region they found attractive.

The proposed novel approach to the analysis of human mobility data involves the reduction of complexity to a single scalar index^2^. This complexity stems from two-dimensional network data, making it difficult to draw clear conclusions from raw origin–destination (OD) flows. Rather than representing intuitive information for each location, OD flow data elucidates the strength of connecting points. In network science literature, centrality measures are standard, and some studies have extended them as real-time mobility data has become more available^3–6^. This research also emphasizes the importance of rich behavioral data on OD flows as network data and recovers the subjective attractiveness of the trip destinations.

This study revisits the conceptual idea of "attractiveness of trip destinations" discussed by Ballou and Pipkin (1977) and Baxter (1979) and develops a simple trip choice model (i.e., choice of trip destination) to measure the attractiveness of trip destinations based on recent theoretical models^7–14^. The standard discrete choice model of interregional mobility predicts that individuals travel to a region where they can obtain the highest utility among the choices. In the literature, Nakajima and Tabuchi (2011) argue that population movements are motivated by the utility gap between regions^15^.

Following Baxter (1979)^8^, the attractiveness of trip destinations is assumed to be related to trip distance, which is also empirically supported by Drezner and Zerom (2023)^16^. For example, a recreational trip to a destination may be an activity that increases individual utility, even if it is a long-distance trip. However, daily commuting decreases individual utility as a mobility cost. Importantly, trip distance affects the perceived attractiveness of trip destinations, varying in terms of the destination and purpose.

The present study advances the human mobility literature by reconsidering the conventional spatial interaction models. Gonzalez-Feliu and Peris-Pla (2017) propose an attractiveness indicator of retail activities based on the gravity equation of trip flows^17^. Although they specify the market potential form under the constant distance decay parameter assumption, we consider the heterogeneous parameters across the regions. Following the idea of the competing destinations model by Fotheringham (1981, 1983)^18,19^, Ito (1986), Yano et al. (2000, 2003) and Babb (2021) estimate heterogeneous distance decay parameters across the origin regions in the conventional gravity equation^20–23^. Although regional attractiveness is measured by the population size parameter to examine whether larger cities offer opportunities for working and living, Ito (1986), Yano et al. (2000, 2003) and Babb (2021) found that the distance decay parameters estimated in the origin-specific gravity equation vary across origin locations, suggesting that some origin regions increase outmigration costs^20–23^. In contrast, this study considers heterogeneous distance decay parameters in the destination-specific gravity equation^24^. Drezner and Zerom (2024) observed that more attractive facilities attract customers from long distances, suggesting that the facility attractiveness decreases the distance decay parameter^16^. Therefore, this study attempts to capture the perceived attractiveness of trip destinations from the distance decay parameter.

Further, this study contributes to the tourism literature by proposing a new way to measure the regional attractiveness of trip destinations. Cracolici and Nijkamp (2009) point out that the dynamic nature of tourist attractiveness should be considered, implying that the attractiveness of trip destinations depends on the trip purposes^25^. Furthermore, Salinas Fernández et al. (2020) emphasize the need to monitor the competitiveness of tourist destinations. Because the tourism industry experienced severe economic deterioration during the pandemic, the monitoring index proposed in this study has important implications for the industry^26^. Wu et al. (2023) investigated the attractiveness of Meeting, Incentive tour, Convention, and Event (MICE) cities in China using spatial network analysis, and the proposed approach in this study provides additional insights into their findings^27^.

The proposed regional attractiveness index offers scale-independent quantification, which is a notable advantage over alternative methods. While measuring annual visitors to a region is crucial for assessing its attractiveness, quantification methods alone enable only comparisons between a region’s past and present states. Understanding the disparities between regions at the same point in time reveals each region’s unique characteristics. However, to draw meaningful comparisons between regions, a quantitative approach must be employed to assess their attractiveness as destinations. This assessment should be conducted from a neutral perspective, independent of any scale or dimensionality. Specifically, the unit of the proposed regional attractiveness index is defined as a dimensionless quantity because it is estimated as an elasticity in the regression.

This study reveals spatial and temporal heterogeneity in the attractiveness of trip destinations. First, it considers how the attractiveness of trip destinations changes for different trip purposes (e.g., commuting to the office and school, recreational trips, business trips, and returning home). For this purpose, this study relies on the Person Trip Survey of the Kansai Metropolitan Area^28^, which provides comprehensive information on various aspects of daily mobility in terms of "why," "from where to where," "who," "when," "how," and "for what purpose." In addition, the estimated regional attractiveness index is visualized on a map as an exploratory spatial data analysis (ESDA) to gain deep insights into its spatial structure. Specifically, this study further applies hot and cold spot analyses using Getis–Ord ${G}_{i}^{*}\left(d\right)$ statistic (See SI Appendix B)^29–31^. Second, this study relies on real-time big data on human mobility based on mobile phone data to focus on the seasonal and tourism factors of trip flows. Based on the high-frequency data, this analysis can reveal how the attractiveness of trip destinations fluctuates seasonally throughout the year.

## Results

### Applied analysis using a person trip survey

Table 1 presents the estimation results of the regional attractiveness index by trip purpose. There is a considerable heterogeneity in the regional attractiveness index. A substantial heterogeneity is observed in the regional attractiveness index. The maximum value that can be assigned to all purposes is $-1.28$, while the minimum value is $-5.75$. The mean of the regional attractiveness index for all purposes is $-2.78$, and the median is $-2.73$. Despite slight variations in the regional attractiveness index across different purposes, analogous results were obtained for quantiles.

**Table 1:** Descriptive statistics of regional attractiveness index from person trip survey. The unit of observation is the municipality. Some municipalities have no value because of an insufficient number of positive trip flows. Obs represents the number of survey zones with estimated regional attractiveness indices. Sig represents the number of survey zones with statistically significant regional attractiveness indices at the 10% level.

Variable	Obs	Sig	Mean	S.D	Min	P25	Median	P75	Max
Regional Attractiveness Index (All purposes)	427	427	− 2.78	0.65	− 5.75	− 3.15	− 2.73	− 2.32	− 1.28
Regional Attractiveness Index (Office)	399	399	− 2.73	0.65	− 5.40	− 3.11	− 2.71	− 2.29	− 1.07
Regional Attractiveness Index (School)	365	365	− 2.78	0.56	− 4.82	− 3.15	− 2.75	− 2.40	− 1.64
Regional Attractiveness Index (Free)	390	390	− 3.03	0.77	− 6.73	− 3.47	− 2.95	− 2.52	− 1.29
Regional Attractiveness Index (Business)	424	424	− 3.04	0.72	− 6.24	− 3.47	− 3.00	− 2.55	− 1.45
Regional Attractiveness Index (Home)	357	357	− 2.17	0.50	− 5.41	− 2.36	− 2.09	− 1.89	− 1.11
Regional Attractiveness Index (Unknown)	377	377	− 2.46	0.67	− 5.75	− 2.83	− 2.33	− 1.97	− 1.18

**Fig. 1:**
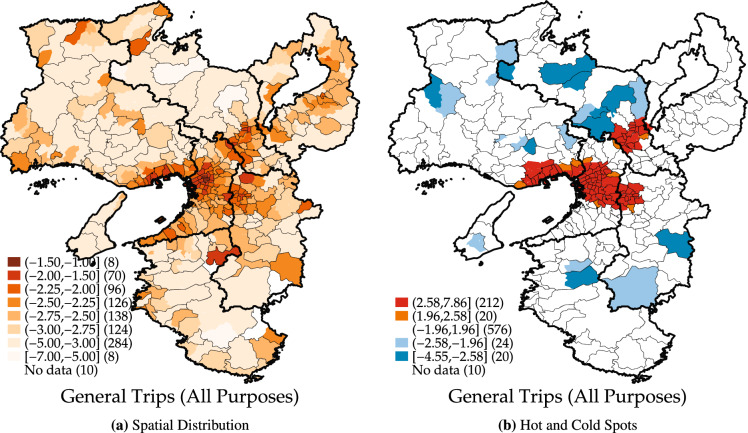
Regional attractiveness index and hot and cold spots of regional attractiveness from person trip survey. The threshold distance $d$ is set to 10 km in calculating the Getis-Ord ${G}_{i}^{*}(d)$ statistics.

Figure 1 visualizes the geographical distributions of the regional attractiveness index for all trip purposes estimated from the Person Trip Survey in the Kansai region of Japan. As illustrated in Fig. 1(a), the metropolitan areas of Osaka, Kobe, and Kyoto exhibit higher values of regional attractiveness index. As illustrated in Fig. 1(b), the results of the hot and cold spot analysis employing the Getis–Ord ${G}_{i}^{*}\left(d\right)$ statistic are presented. The hot spot areas of regional attractiveness are detected over the Osaka, Kobe, Nara, and Kyoto. An interesting feature is that the hot spot in Kyoto city is located away from that centered on Osaka city. to the index of regional attractiveness estimated from Person Trip Survey.

**Fig. 2:**
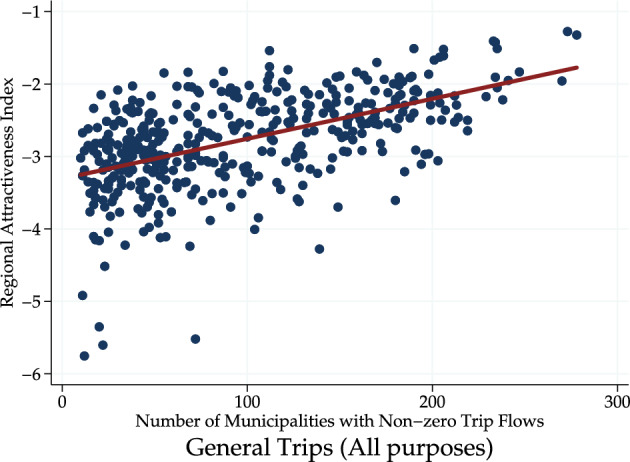
Regional attractiveness index and number of municipalities with non-zero trip flows from person trip survey. The number of municipalities with non-zero trip flows (i.e., the number of direct connections a node has within a network) is conceptually equivalent to the degree centrality, which is one of the centrality measures used in network analysis.

Figure 2 illustrates the relationship between the regional attractiveness index and the number of municipalities with non-zero trip flows (i.e., degree centrality within the trip network). Figure 2 shows that municipalities with high degree centrality are always highly attractive. On the other hand, municipalities with a high regional attractiveness index do not necessarily have a high degree of centrality. This finding suggests that direct connections with other municipalities are a factor that explains high regional attractiveness.

**Fig. 3:**
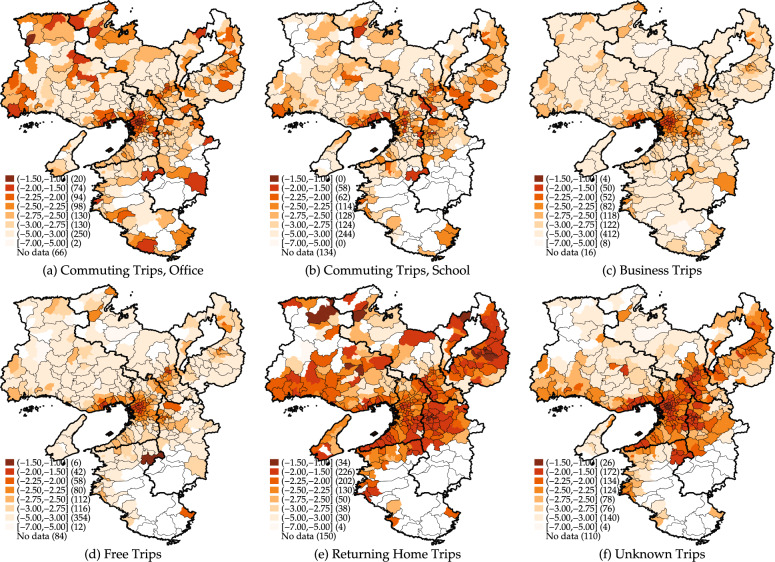
Regional attractiveness index by trips purpose from person trip survey.

The trips for all purposes are further disaggregated to consider how trip purposes affect regional attractiveness in Fig. 3. Figure 3(a) shows the spatial distribution of regional attractiveness based on people’s commuting trips to their workplaces. Although downtown areas in larger cities still show higher attractiveness, some suburban areas attract people from a region-wide perspective as a regional core. Figure 3(b) displays the spatial distribution of regional attractiveness based on commuting trips to school. The regional attractiveness estimated from school trips is more diversified in metropolitan areas. Rural areas show missing, suggesting limited access to schools from outside. Figure 3(c) and 3(d) show the spatial distributions of regional attractiveness based on free and business trips, respectively. In both cases, urban areas with hub stations tended to show higher attractiveness because they widely attracted people. Figure 3(e) illustrates the spatial distribution of regional attractiveness considering the trips to returning home. Unlike commuting to the office, free, and business trips, returning home trips reveal the attractiveness of residence location. For example, Shiga and Nara prefectures attract migrants who commute to the central business district of the Kansai region. Therefore, these regions show higher regional attractiveness as living places. Figure 3(f) shows the spatial distribution of regional attractiveness according to unknown (unclassified) trips. Although the trip purpose was unknown from the data, the regional attractiveness estimated that this type of travel would likely be concentrated in core urban areas.

**Fig. 4:**
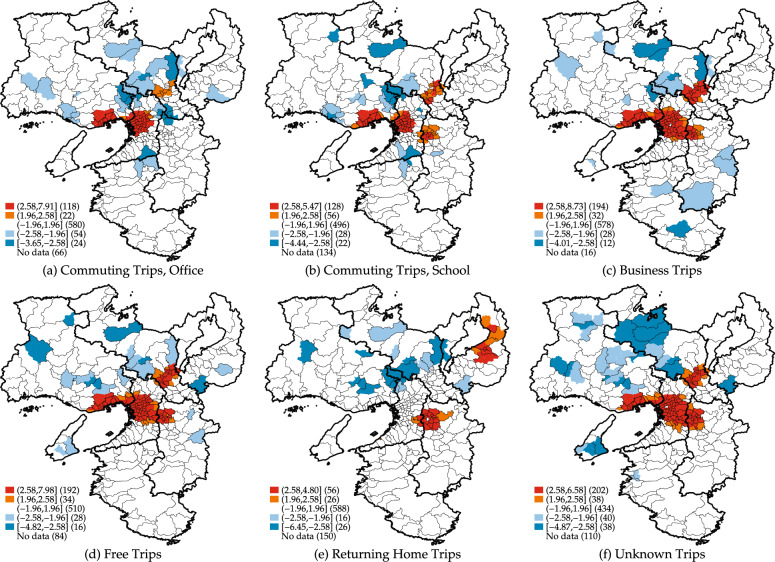
Hot and cold spots of regional attractiveness by trip purpose from person trip survey. The threshold distance $d$ is set to 10 km in calculating the Getis-Ord ${G}_{i}^{*}(d)$ statistics.

Figure 4 visualizes the hot and cold spots of the regional attractiveness index in Fig. 3. As expected, the largest cities in the Kansai region, namely Kyoto, Osaka, and Kobe cities, tend to show hot spots of regional attractiveness, as stated for all trip purposes. However, important insights into regional attractiveness are also found. For example, Fig. 3(e) shows that there are hot spots of regional attractiveness for returning home trips in Shiga and Nara prefectures, suggesting that these areas are more attractive as a residential place, given their accessibility to public transportation.

### Applied analysis using mobile phone data

Table 2 presents the monthly regional attractiveness indices estimated from the mobile phone data from September 2015 to August 2016 in the entire area of Japan. The regional attractiveness index shows a greater variation across municipalities. The mean of the regional attractiveness index ranges from $-2.4$ and $-2.7$ on weekdays and weekends/holidays in the study period. The median also takes a similar value. The maximum value of the regional attractiveness index generally takes a value smaller than one.

**Table 2:** Descriptive statistics of regional attractiveness index from mobile phone data. The unit of observation is the municipality. Some municipalities have no value because of an insufficient number of positive trip flows. The data above are based on the estimation results of gender (total) and age group (total). Obs represents the number of municipalities with estimated regional attractiveness indices. Sig represents the number of municipalities with statistically significant regional attractiveness indices at the 10% level.

Variable	Obs	Sig	Mean	S.D	Min	P10	Median	P90	Max
*Weekdays* Regional Attractiveness Index (Sep 2015)	1808	1808	− 2.66	0.65	− 6.12	− 3.04	− 2.60	− 2.23	− 0.91
Regional Attractiveness Index (Oct 2015)	1816	1816	− 2.66	0.67	− 11.18	− 3.06	− 2.62	− 2.23	− 0.67
Regional Attractiveness Index (Nov 2015)	1806	1806	− 2.71	0.63	− 7.43	− 3.08	− 2.68	− 2.27	− 0.69
Regional Attractiveness Index (Dec 2015)	1822	1822	− 2.47	0.57	− 5.19	− 2.80	− 2.42	− 2.09	− 0.45
Regional Attractiveness Index (Jan 2016)	1773	1773	− 2.69	0.69	− 7.68	− 3.07	− 2.64	− 2.24	− 0.58
Regional Attractiveness Index (Feb 2016)	1779	1779	− 2.72	0.67	− 8.25	− 3.08	− 2.65	− 2.28	− 0.63
Regional Attractiveness Index (Mar 2016)	1801	1801	− 2.64	0.64	− 9.33	− 3.02	− 2.58	− 2.21	− 0.83
Regional Attractiveness Index (Apr 2016)	1793	1792	− 2.69	0.67	− 6.32	− 3.08	− 2.64	− 2.22	− 0.52
Regional Attractiveness Index (May 2016)	1802	1802	− 2.68	0.63	− 7.35	− 3.03	− 2.62	− 2.24	− 0.95
Regional Attractiveness Index (Jun 2016)	1812	1811	− 2.72	0.65	− 6.65	− 3.10	− 2.68	− 2.25	− 1.01
Regional Attractiveness Index (Jul 2016)	1815	1815	− 2.71	0.65	− 6.64	− 3.09	− 2.66	− 2.25	− 1.19
Regional Attractiveness Index (Aug 2016)	1851	1851	− 2.36	0.53	− 9.36	− 2.66	− 2.31	− 2.00	− 0.94
*Weekends*/*Holidays* Regional Attractiveness Index (Sep 2015)	1796	1795	− 2.49	0.54	− 5.16	− 2.80	− 2.44	− 2.13	− 0.87
Regional Attractiveness Index (Oct 2015)	1772	1772	− 2.62	0.59	− 6.30	− 2.95	− 2.58	− 2.23	− 1.00
Regional Attractiveness Index (Nov 2015)	1772	1772	− 2.62	0.59	− 8.91	− 2.96	− 2.59	− 2.24	− 1.00
Regional Attractiveness Index (Dec 2015)	1690	1689	− 2.74	0.65	− 5.86	− 3.11	− 2.69	− 2.32	− 1.03
Regional Attractiveness Index (Jan 2016)	1808	1808	− 2.34	0.50	− 5.30	− 2.60	− 2.29	− 2.01	− 0.98
Regional Attractiveness Index (Feb 2016)	1686	1686	− 2.73	0.66	− 7.53	− 3.10	− 2.66	− 2.31	− 1.08
Regional Attractiveness Index (Mar 2016)	1735	1735	− 2.60	0.59	− 5.76	− 2.94	− 2.56	− 2.21	− 0.94
Regional Attractiveness Index (Apr 2016)	1749	1748	− 2.59	0.61	− 7.75	− 2.94	− 2.53	− 2.16	− 0.28
Regional Attractiveness Index (May 2016)	1819	1818	− 2.45	0.52	− 8.84	− 2.73	− 2.41	− 2.10	− 0.69
Regional Attractiveness Index (Jun 2016)	1736	1736	− 2.70	0.62	− 5.99	− 3.04	− 2.64	− 2.28	− 1.12
Regional Attractiveness Index (Jul 2016)	1793	1793	− 2.58	0.59	− 9.22	− 2.91	− 2.54	− 2.20	− 1.08
Regional Attractiveness Index (Aug 2016)	1827	1827	− 2.32	0.49	− 9.17	− 2.60	− 2.29	− 2.00	− 0.96

**Fig. 5:**
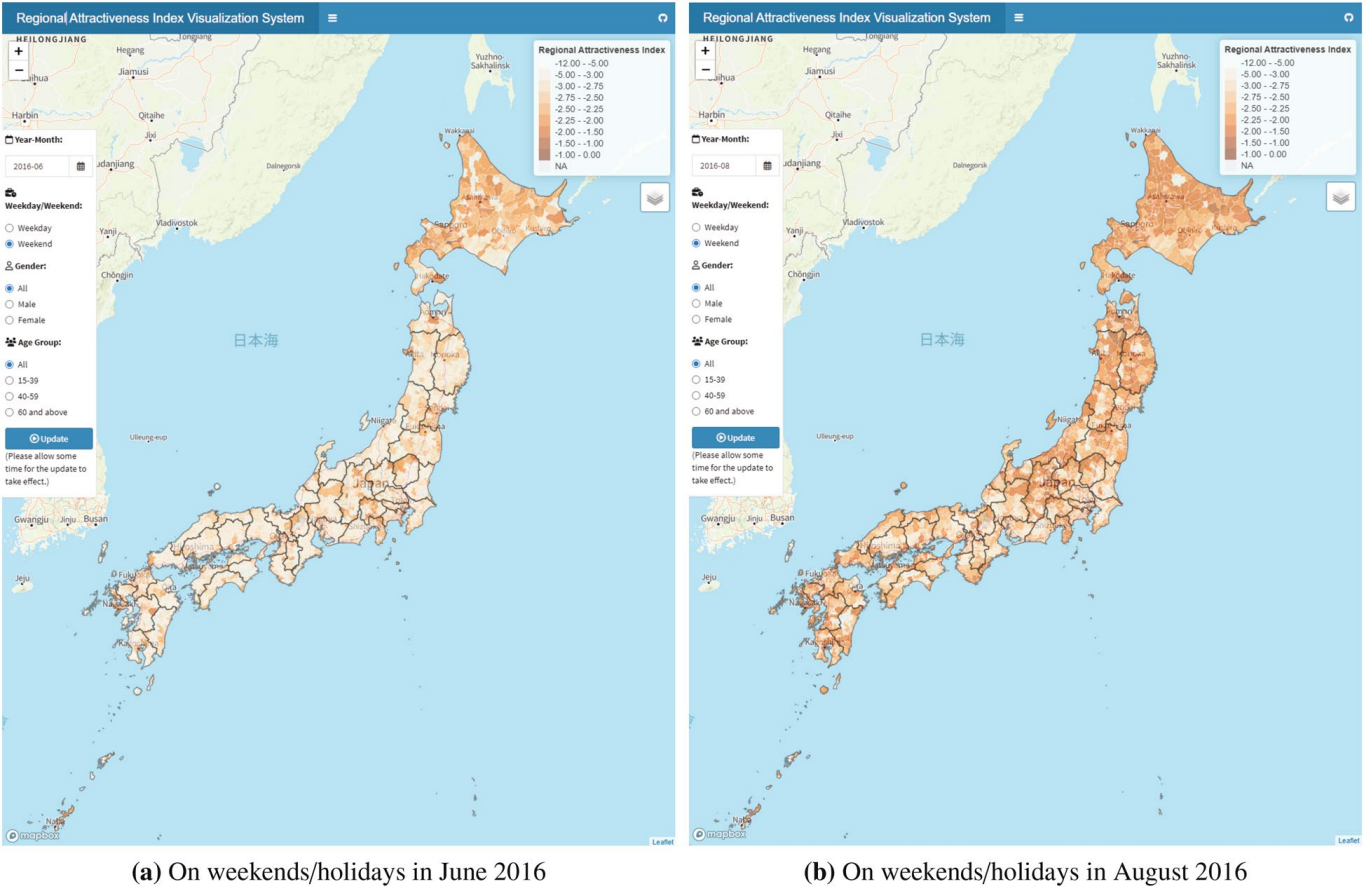
Spatial distribution of the regional attractiveness index estimated using mobile phone data. The distribution of the regional attractiveness index varies seasonally.

Figure 5 visualizes the geographic distributions of the regional attractiveness index, comparing them on weekends/holidays between June 2016 and August 2016. A comparison between Fig. 5(a) and 5(b) provides an interesting result regarding seasonal factors. Whereas rural municipalities with a high regional attractiveness index were concentrated in the central business district in June 2016, rural municipalities showed higher values of the regional attractiveness index in August 2016 because of the summer vacation season. These findings suggest that the attractiveness of trip destinations dynamically fluctuates throughout the year. Exploiting the advantage of the high-frequency big data, this study estimates the regional attractiveness indices across months, day type (weekday and weekend/holiday), gender (total, male, and female), and age group (total, 15–39, 40–59, and 60 and above). All results are shown on the web app.

**Fig. 6:**
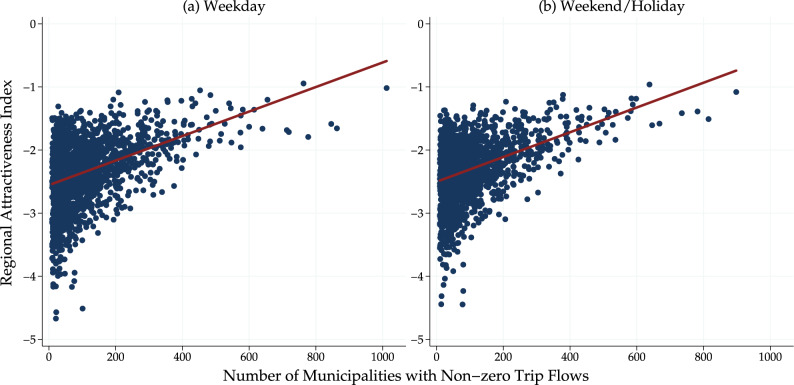
Regional attractiveness index and number of municipalities with non-zero trip flows from mobile phone data. The number of municipalities with non-zero trip flows (i.e., the number of direct connections a node has within a network) is conceptually equivalent to the degree centrality, which is one of the centrality measures used in network analysis.

Figure 6 illustrates the relationship between the regional attractiveness index and the degree centrality within the trip network. Specifically, Fig. 6 shows that municipalities with high degree centrality are always highly attractive. However, municipalities with a high regional attractiveness index do not necessarily have a high degree of centrality, suggesting that degree centrality is one factor that explains regional attractiveness, but there are also other factors that increase the regional attractiveness.

**Fig. 7:**
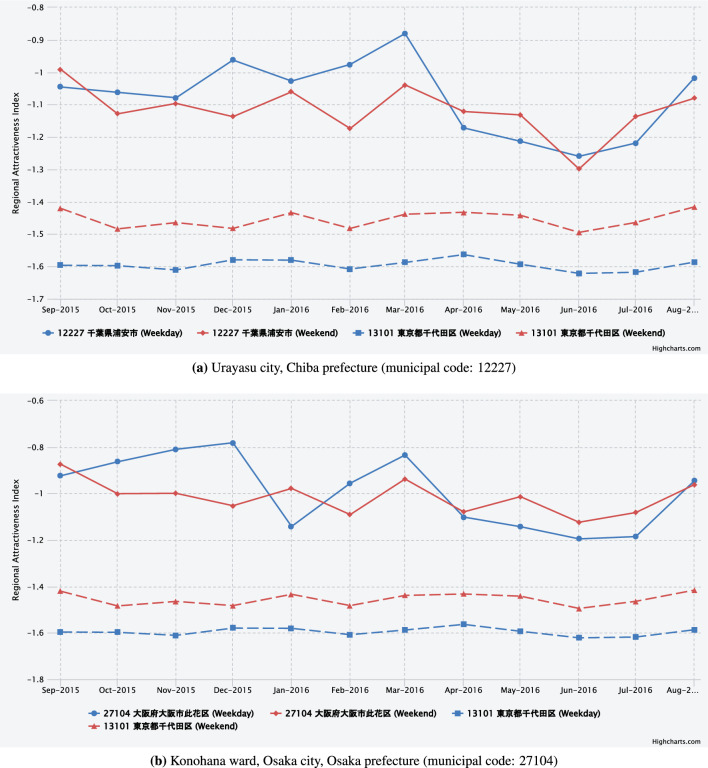
Trend of the regional attractiveness index estimated from the mobile phone data in municipalities with famous theme and amusement parks. The dashed lines show the trends of the regional attractiveness index in Chiyoda ward, Tokyo (municipal code: 13,101), which is used as a benchmark municipality.

**Fig. 8:**
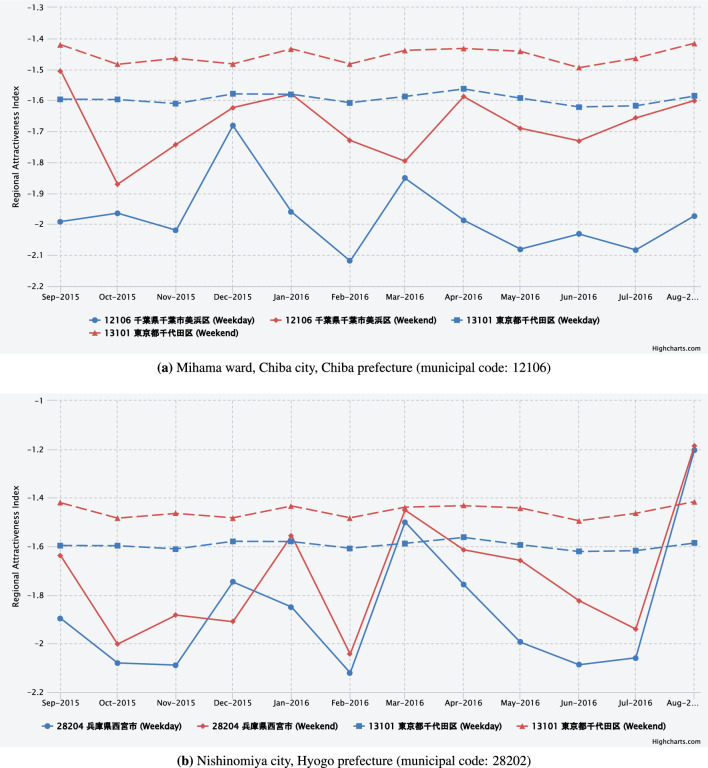
Trends of the regional attractiveness index estimated from the mobile phone data in municipalities hosting major events or sporting events. The dashed lines show the trends of the regional attractiveness index in Chiyoda ward, Tokyo (municipal code: 13,101), which is used as a benchmark municipality.

**Fig. 9:**
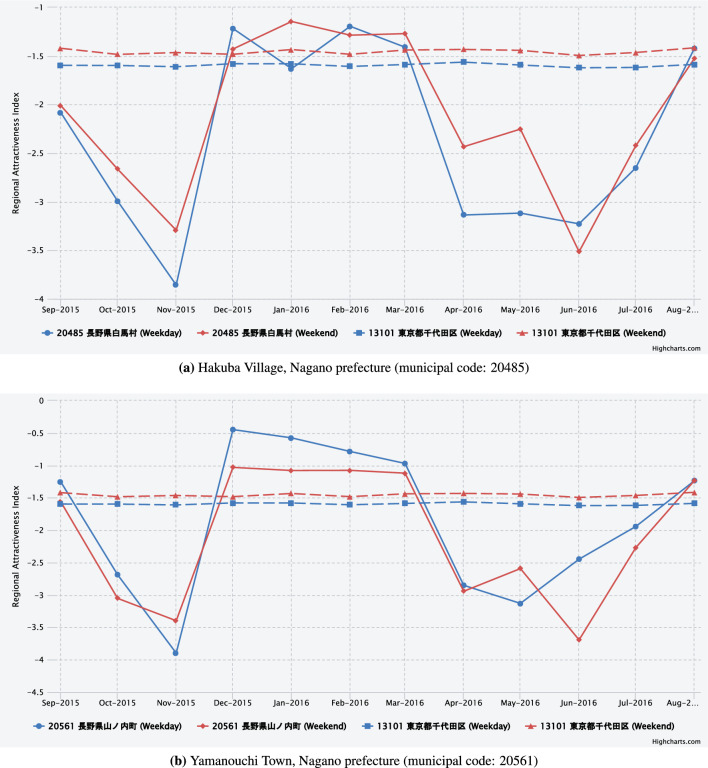
Trends of the regional attractiveness index estimated from the mobile phone data in mountainous municipalities famous for its ski resorts. The dashed lines show the trends of the regional attractiveness index in Chiyoda ward, Tokyo (municipal code: 13,101), which is used as a benchmark municipality.

Figures 7, 8 and 9 illustrate the seasonal trends in the regional attractiveness index with a focus on the different types of cities.

Figure 7 focuses on the municipalities with widely known amusement and theme parks. Figure 7(a) shows the regional attractiveness index for Urayasu city, Chiba prefecture. The index remains around $-1$ throughout the entire period, a value that is among the highest recorded for municipalities. The presence of Tokyo Disney Resort in a city is one potential factor contributing to its high attractiveness as a trip destination. Specifically, the index demonstrates an upward trend on weekdays during the month of March, suggesting that the city experiences a significant influx of visitors from various municipalities of Japan during the spring break season, which corresponds with school promotions and graduations. When analyzed by gender and age group, the city is found to be more attractive to women than to men and to those under 60 years old than to those over 60 (See web app). Furthermore, the study found that Urayasu city was visited by residents from more than half of all municipalities nationwide, thereby further substantiating its high regional attractiveness.

Figure 7(b) shows the regional attractiveness index for Konohana ward, Osaka city, Osaka prefecture, where the world-renowned theme park Universal Studios Japan is located. The index has remained around $-$ 1 throughout the entire period, indicating that the area has extremely high regional attractiveness as a destination, similar to Urayasu city in Chiba prefecture. Moreover, it is consistently ranked among the top municipalities nationwide throughout the year. As the host city for the 2025 Osaka-Kansai World Expo, it will be interesting to observe how the regional attractiveness index for Osaka city’s Konohana ward evolves in the coming years.

Figure 8 focuses on the municipalities in which regular, large-scale events take place throughout the year. Figure 8(a) shows the regional attractiveness index for Mihama ward, Chiba city, Chiba prefecture. The index reveals a significant disparity between weekdays and weekends/holidays. During weekdays, the index maintains a consistent level of approximately $-2$. However, during weekends/holidays, it exhibits a greater degree of variability, fluctuating between $-1.6$ and $-1.7$. A notable factor contributing to the elevated level of attractiveness observed in the Mihama ward of Chiba city during weekends is the presence of Makuhari Messe, a substantial event venue. A significant number of events are scheduled on weekends and holidays, drawing visitors from various municipalities across Japan.

Figure 8(b) shows the regional attractiveness index for Nishinomiya city, Hyogo prefecture. The index fluctuated between $-1.8$ and $-2$. Moreover, in August, the index exhibited a pronounced surge to approximately $-1.2$, indicating the presence of a particular factor that enhanced the regional attractiveness. This phenomenon is likely attributable to the influence of the prominent Summer Koshien (National High School Baseball Championship), suggesting that the proposed method captures seasonal and event-related factors as indicators of regional attractiveness.

Figure 9 focuses on the mountainous municipalities. Figure 9(a) shows the regional attractiveness index of Hakuba village, Nagano prefecture, one of the renowned ski resort areas. The index fluctuates between $-1$ and $-1.5$ during the winter months (December to March). It is one of the villages that hosted the 1998 Winter Olympics in Nagano prefecture. The index also demonstrates a marked increase during the summer months, coinciding with the peak seasons for mountain climbing, hiking, and trekking activities.

Figure 9(b) shows the regional attractiveness index of Yamanouchi town, Nagano prefecture. Similar to the Hakuba village, the index fluctuates between -0.5 and -1 during the winter months (December to March). Yamanouchi town is a nationally renowned resort area known for Shiga Highlands and Yudanaka Shibu Onsenkyo, and it is also popular as a ski resort in winter. On the other hand, during the off-season, the index drops to around -3, indicating that the region experiences significant fluctuations throughout the year. Despite its small population, Yamanouchi town demonstrates that it can possess charm rivaling that of large cities during certain seasons. Many other municipalities in mountainous areas exhibit similar characteristics.

## Discussion

Revisiting the original idea of Baxter (1979)^8^, this study discussed that a destination-fixed gravity equation provides a simple framework for estimating the heterogeneous attractiveness of each trip destination. Given that individuals’ choice of trip destination is based on utility maximization, this study assumed that trip flows include fundamental information on the attractiveness of trip destinations. The novel approach proposed in this study successfully addressed the complexity of using human mobility data by reducing the dimension to a single scalar. The ESDA, using the estimated regional attractiveness index, contributed to understanding the spatial structure of the attractiveness of trip destinations.

This first empirical analysis based on the 2010 Person Trip Survey in the Kansai region of Japan provided insights into how regional attractiveness depends on different mobility purposes. The second empirical analysis based on the mobile phone data also revealed the attractiveness of trip destinations fluctuates seasonally throughout the year. These findings show that the proposed approach using interregional trip flows captured the extent to which trip destinations attract people from a region-wide perspective a scalar index of regional attractiveness.

The empirical results of this study have significant implications for regional attractiveness. While urban centers are generally considered attractive, the regional attractiveness of a given area is not constant over time. Additionally, certain rural regions have been observed to exhibit elevated levels of the regional attractiveness index. Therefore, the attractiveness of a trip destination is contingent upon the purpose of the trip and seasonal factors. This finding aligns with the observations reported by Zhong et al. (2015), who found variability in the mobility patterns^32^.

This study made an important contribution by bridging the gap between academic research and the development of policy. During the Coronavirus disease (COVID-19) pandemic, a critical policy strategy was to monitor the key performance indicators of the local economic situation on a daily basis, conceptualizing this as a vital sign. However, local economic performance indicators are generally unavailable as high-frequency data because research based on large sample surveys takes a long time to yield meaningful results. Consequently, this study investigated the capacity of big data to enhance the prevailing approach to policymaking and evaluation^33–35^. As big data on human mobility based on smartphones becomes ubiquitous, our proposed approach can be applied to the data to secure valuable insights for local economic revitalization and inform policymaking.

It must be acknowledged that the present study is not without its limitations. While the regional attractiveness index plays a pivotal role as a monitoring indicator of local economic vitality and provides a novel perspective for the ESDA, it is also imperative to explore the factors that contribute to regional attractiveness. For instance, regions equipped with hub stations consistently attract passengers from neighboring regions. The occurrence of local population events has been demonstrated to result in an increase in temporal trips, thereby inducing a temporal increase in regional attractiveness. In such cases, the synthetic control method can serve as a valuable tool for evaluating the causal impact on the attractiveness of a trip destination. Consequently, conducting a statistical analysis to examine the factors that contribute to regional attractiveness is imperative to comprehensively understand local economic performance. These challenges must be addressed in future research endeavors.

## Method

### Dataset for person trip survey

**Fig. 10:**
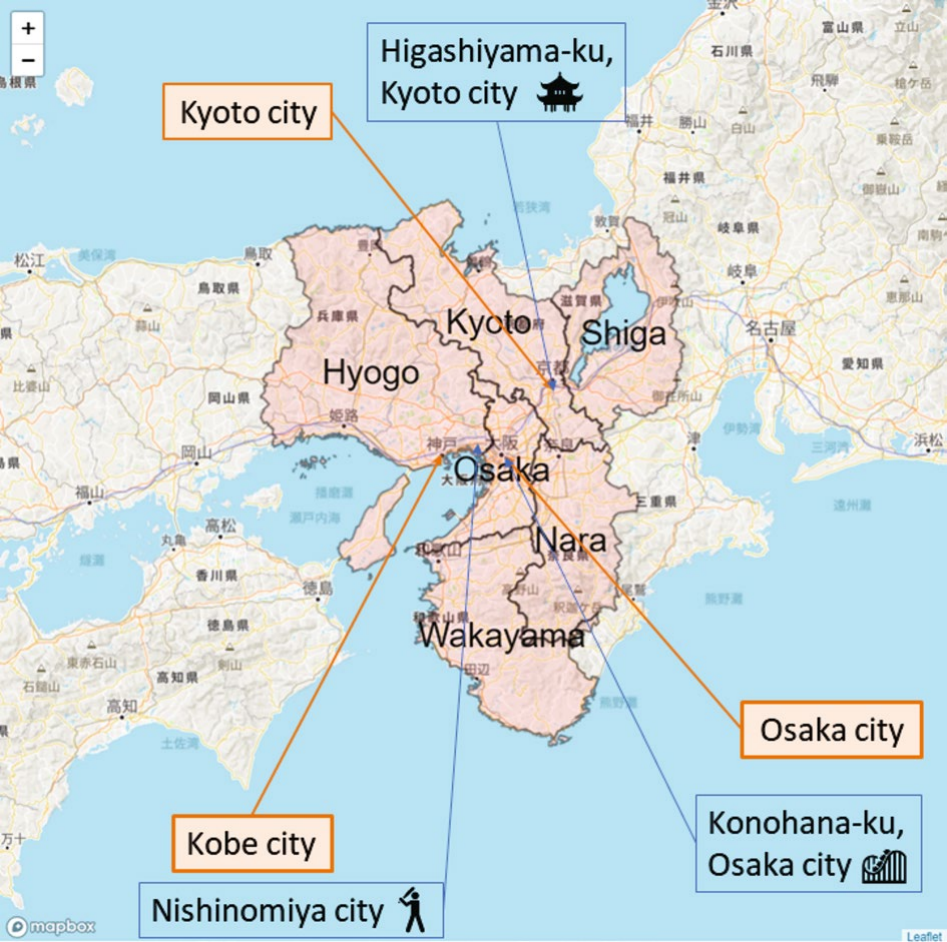
Kansai region of Japan includes Shiga, Kyoto, Osaka, Hyogo, Nara, and Wakayama prefectures. The 2010 Person Trip Survey conducted in the Kansai region covers these six prefectures. The locations of some cities discussed in this paper are shown on the map. Higashiyama ward, Kyoto city has many historical tourist resources and attract many visitors throughout the year. Konohana ward, Osaka city has one of the most popular amusement parks, the Universal Studios Japan. High school baseball at Koshien Staduim in Nishinomiya city is one of the most popular sport evens in Japan.

The first dataset of the interregional trip flows is the 2010 Person Trip Survey conducted in the Kansai region (Shiga, Kyoto, Osaka, Hyogo, Nara, and Wakayama prefectures)^28^. Figure 10 shows the survey area in the Kansai region. The survey was conducted from October to November 2010 to capture daily human mobility based on factors such as the purpose of the trip, origin and destination, individuals involved, time and mode of transportation, and the trip’s intent. Such detailed information on human mobility is essential for gauging the attractiveness of trip destinations, as regional attractiveness is not invariant but depends on the trip purpose.

Person Trip Surveys are generally conducted every ten years in each urban area across Japan. The first survey in the Kansai region was carried out in 1970, and the fifth was conducted in 2010. Publicly available data are aggregated at the survey unit level, which is more disaggregated than the municipal unit level. While there are 245 municipalities and wards designated by government ordinance in the Kansai region, the 2010 Person Trip Survey covered 432 survey zones within them. This geographical disaggregation generally improves spatial analysis. This study uses a dataset of weekday trips, while the 2010 Person Trip Survey also surveyed weekend trips.

Table 3 presents the descriptive statistics on trip flows and bilateral distances between the survey areas concerning the positive trip flows. It is important to note that the original data set contains numerous zero trip flows. For instance, out of 186,192 ($=432\times 432-432$) flows with respect to all purposes, 40,767 (approximately 22%) are classified as positive. Therefore, the descriptive statistics are calculated except zero flows. The mean of the number of trips for all purposes is 371.50, while the median is 75. The mean is found to be greater than the 75th percentile. This suggests that the distribution of trips is extremely skewed to the right.

**Table 3:** Descriptive statistics of trip flows from person trip survey. The unit of observation is the trip flow between survey zones. The total number of interregional trip flows is 186,192 (= 432 × 432 − 432), and the gravity equation is estimated after excluding the intra-municipal flows. Trip flows within the diameter of each zone are excluded as internal trip flows. Obs represents the positive trip flows between survey zones.

Variable	Obs	Mean	S.D	Min	P25	Median	P75	Max
Number of Trips (All purposes)	40,767	371.50	1154.51	1	32	76	251	36,938
Number of Trips (Commuting, office)	23,468	185.46	552.85	1	26	49	128	15,189
Number of Trips (Commuting, school)	17,956	168.37	508.56	1	33	56	131	13,892
Number of Trips (Free)	21,359	167.78	414.54	1	27	53	135	10,761
Number of Trips (Business)	28,003	230.69	697.25	1	30	63	169	23,821
Number of Trips (Home)	20,714	186.08	444.65	1	30	58	151	7,815
Number of Trips (Unknown)	30,071	285.74	644.07	1	34	82	245	14,603
Distance (km)	184,932	80.53	43.36	1.88	46.53	76.09	109.48	261

This study considers seven types of trips for different purposes (all purposes, commuting to office, commuting to school, free, business, returning home, and unknown). Note that returning home trip indicates the trip flow from locations where individuals stay outside to their residential locations. Therefore, the trip destination is the residential location, not the workplace and school locations.

The travel distance was determined by measuring the great circle distance from the longitude and latitude of the reference point of the survey zone. The study establishes the centroid of each polygon as a reference point because the geographical discrepancy between the centroid of the polygons and the location of the central business districts is relatively small for the survey zones of the Person Trip Survey. The great circle distances were calculated based on Vincenty’s formula, using Stata’s geodist command^36^.

### Dataset for mobile phone data

The second dataset on the interregional trip flows is the Mobile Spatial Statistics of NTT DOCOMO^37^, which offers big data on interregional mobility obtained by geospatial information technology, based on the locational information of mobile phone users. The Mobile Spatial Statistics is based on NTT DOCOMO’s mobile phone users, covering approximately 40% of the total population in Japan. Unlike the traditional survey, the Mobile Spatial Statistics estimates population distribution with fluctuation over time, 24 h a day, and 365 days a year. The locational information is obtained from the base stations in the mobile terminal network (not mobile phone GPS).

This study employs the monthly data on inter-municipal flows from September 2015 to August 2016 by the day of the week (weekday or weekend/holiday). The dataset used in this study was freely available from the Regional Economy and Society Analyzing System (RESAS), a web application developed by the Headquarters for Overcoming Population Decline and Vitalizing Local Economy in Japan at the Prime Minister’s Office^38^. Detailed information on inter-municipal flows was available by gender, age, year, month, day of the week (weekdays and weekends), and time of day (4 am, 10 am, 2 pm, 8 pm). In this study, the interregional trip flows are based on the residential municipalities and the municipalities where mobile phone users stayed at 2 pm.

Although we focus only on the Kansai region in the Person Trip Survey, the Mobile Spatial Statistics covers the entire area of Japan, covering 1,896 municipalities and wards of cities designated by government ordinance. The cities designated by government ordinance are cities with populations greater than 500,000 and are designated by the government under Article 252–19 of the Local Autonomy Act. In November 2023, there are 20 cities (Sapporo, Sendai, Saitama, Chiba, Yokohama, Kawasaki, Sagamihara, Niigata, Shizuoka, Hamamatsu, Nagoya, Kyoto, Osaka, Sakai, Kobe, Okayama, Hiroshima, Fukuoka, Kitakyushu, and Kumamoto) in Japan.

Table 4 presents the descriptive statistics on inter-municipal human mobility flows and bilateral distances regarding weekdays and weekends/holidays. As previously mentioned, it is important to note that the original data set contains a substantial number of zero trip flows. For instance, out of 3,592,920 ($=\mathrm{1,896}\times \mathrm{1,896}-\mathrm{1,896}$) flows, only 197,897 (approximately 5.5%) were classified as positive on weekdays in August 2015. Consequently, the calculation of descriptive statistics is conducted, with the exception of zero flows. The mean of the number of trips on weekdays in September of 2015 was 213.52, while the median was 43. The mean value exceeds the 75th percentile. As previously mentioned, this finding indicates that the distribution of trips is significantly right-skewed. The minimum number of trips flows is set at 10, as stipulated by the Mobile Spatial Statistics privacy protection protocol.

**Table 4:** Descriptive statistics of inter-municipal trip flows from mobile phone data. The unit of observation is the trip flow between municipalities. The total number of inter-municipal flows is 3,592,920 (= 1,896 × 1,896 − 1,896), and the gravity equation is estimated after excluding the intra-municipal flows. Obs represents the positive trip flows between municipalities. The data above are based on the estimation results of gender (total) and age group (total).

Variable	Obs	Mean	S.D	Min	P25	Median	P75	Max
*Weekdays* Number of Trips (Sep 2015)	151,636	213.52	824.06	10	14	43	126	40,577
Number of Trips (Oct 2015)	147,531	227.20	855.01	10	17	48	137	41,348
Number of Trips (Nov 2015)	137,821	239.39	884.98	10	20	51	143	41,580
Number of Trips (Dec 2015)	172,353	192.52	732.99	10	15	46	125	38,371
Number of Trips (Jan 2016)	145,901	218.85	828.19	10	15	45	132	40,299
Number of Trips (Feb 2016)	136,871	228.00	855.62	10	16	46	135	41,099
Number of Trips (Mar 2016)	156,936	204.57	798.17	10	14	43	124	41,010
Number of Trips (Apr 2016)	157,875	212.49	824.87	10	14	42	127	41,953
Number of Trips (May 2016)	141,099	233.67	869.71	10	19	49	142	42,143
Number of Trips (Jun 2016)	135,054	243.84	901.00	10	20	52	146	42,656
Number of Trips (Jul 2016)	135,665	239.43	886.91	10	20	50	143	42,467
Number of Trips (Aug 2016)	197,897	169.28	669.95	10	14	42	116	38,284
*Weekends*/*Holidays* Number of Trips (Sep 2015)	159,154	170.47	522.24	10	17	50	136	26,299
Number of Trips (Oct 2015)	135,107	190.43	580.12	10	19	53	145	27,731
Number of Trips (Nov 2015)	135,905	189.37	573.41	10	20	54	146	27,335
Number of Trips (Dec 2015)	118,699	201.60	619.92	10	17	51	146	28,566
Number of Trips (Jan 2016)	175,731	150.68	460.34	10	14	46	127	23,595
Number of Trips (Feb 2016)	118,832	194.24	587.63	10	17	51	143	26,951
Number of Trips (Mar 2016)	139,848	181.71	564.93	10	15	48	137	27,291
Number of Trips (Apr 2016)	146,847	177.86	555.91	10	15	47	135	27,942
Number of Trips (May 2016)	163,291	168.44	514.04	10	18	52	137	27,339
Number of Trips (Jun 2016)	120,203	203.96	612.57	10	20	54	152	28,946
Number of Trips (Jul 2016)	139,588	182.61	557.27	10	19	53	140	27,454
Number of Trips (Aug 2016)	180,333	149.48	454.96	10	14	45	125	24,352
Distance (km)	3,592,920	590.96	425.95	0.36	251	499	858	2,961

The Mobile Spatial Statistics provides monthly data on inter-municipal human mobility flows, allowing for a dynamic trend of the regional attractiveness index. For example, seasonal events in some municipalities attract more people from outside municipalities. The proposed method detects changes in inter-municipal human mobility flows.

Bilateral distances are calculated between the most densely populated areas within municipalities in the Mobile Spatial Statistics. The centroid of a polygon is usually used to calculate the distance between zones. However, this method can be misleading if the centroid is located far from a central business district or a major residential area. Unlike the Person Trip Survey, the reference point for municipalities is determined by population concentration using 2020 Population Census data disaggregated by geography in mobile phone data analysis.

### Modeling choice of trip destination and regional attractiveness

The choice of trip destination is considered by a discrete choice model based on the random utility model^9–14^. Suppose that there are $N$ locations in the economy. Each individual who resides in the location $i$ decides to travel to location $j$ for the purpose $m$. Individuals choose one of $N$ trip destinations to maximize their utility. The total utility of each individual, ${\widetilde{V}}_{ijm}$, is defined as follows:

1 $${\widetilde{V}}_{ijm}={V}_{ijm}{b}_{ijm},$$

where ${V}_{ijm}$ is the deterministic utility and ${b}_{ijm}$ > 1 is a stochastic utility including amenities related to locations $i$ and $j$. The deterministic utility ${V}_{ijm}$ is defined as the indirect utility obtained from the utility maximization, as defined below.

The preferences of individuals are defined over the amenity consumption in trip destination $j$, ${A}_{j}$, the consumption of non-trip goods and services in residential location $i$, ${C}_{i}$, and the discount factors of objective and subjective trip costs from locations $i$ to $j$, ${f}_{O}({D}_{ij})$ and ${f}_{S, m}\left({D}_{ij}\right)$, where ${D}_{ij}$ is the bilateral distance between locations $i$ and $j$.

The utility function is assumed to take a Cobb-Douglass form as follows:

2 $${U}_{ijm}\left({A}_{jm},{C}_{i}\right)=\dfrac{1}{{\mu}^{\mu}{\left(1-{\mu}\right)}^{1-{\mu}}} \left[{f}_{O}\left({D}_{ij}\right){f}_{S,m}\left({D}_{ij}\right)\right] {A}_{j}^{\mu}{C}_{i}^{1-{\mu}},$$

where $\mu$ is the expenditure share for amenity and the second term on the right-hand side.

The attractiveness of trip destinations can be interpreted as a factor of the individuals’ utility. This study introduces the discount factors of objective and subjective trip costs, ${f}_{O}({D}_{ij})$ and ${f}_{S,m}({D}_{ij})$, respectively, The range of the objective and subjective discount factors is from zero to one. The objective trip costs are common among trips, whereas the subjective trip costs vary between trips in terms of trip purposes. When ${f}_{S,m}\left({D}_{ij}\right)=1$, this is a standard assumption of the trip choice model. The attractiveness of trip destinations must be high as the discount factor of subjective trip costs approaches to one because it means individuals obtain higher utility from the trip. Based on this assumption, this study proposes an empirical approach to estimating the index of regional attractiveness based on interregional trip flows.

The budget constraint is expressed as follows:

3 $${P}_{{A},{j}}{A}_{j}+{P}_{{C},{i}}{C}_{i}={I}_{i},$$

where ${P}_{A,j}$ is the price of amenity in trip destination $j$, ${P}_{C,i}$ is the price of good in residential location $i$, and ${I}_{i}$ is the individual’s income in location $i$.

Utility maximization yields the demand functions for amenities in location $j$ and non-trip goods and services in location $i$, and substituting them into the utility function in Eq. (2), we have the indirect utility function as follows:

4 $${V}_{ijm} = \dfrac{{I}_{i}}{{P}_{A,j}^{\mu}{P}_{C,i}^{1-{\mu}}} \left[{f}_{O} \left({D}_{ij} \right){f}_{S,m}\left(D_{ij}\right) \right]$$

Next, a stochastic utility component is assumed to be drawn from an independent Fréchet distribution. The cumulative distribution function of Fréchet distribution, ${F}_{ijm}\left(b\right)$, is expressed as follows:

5 $${F}_{ijm} \left(b\right)={\mathrm{exp}}\left(-{B}_{im}{B}_{jm}{b}^{-\alpha} \right), \; {B}_{im}>0,\; {B}_{jm}>0, \; \alpha>1,$$

where ${B}_{im}$ is a scale parameter that determines average utility derived from the residential location, ${B}_{jm}$ is the scale parameter that determines the average utility derived from trip destination $j$, and $\alpha$ is a shape parameter^9,10^. In the existing literature, Crozet (2004) and Kondo and Okubo (2015) consider a theoretical framework in which stochastic amenities are introduced additively, called additive random utility models^39,40^.

An individual chooses a choice that maximizes utility among all choices for the trip purpose $m$. The assumption of Fréchet distribution yields the probability of trip for the purpose $m$ from location $i$ to location $j$ as follows:

6 $${\pi}_{ijm} = \dfrac{{B}_{jm}{P}_{A,j}^{-\mu \alpha}{\left[{f}_{O}\left({D}_{ij}\right){f}_{S,m}\left({D}_{ij}\right)\right]}^{\alpha}}{{\sum}_{k=1}^{N}{B}_{km}{P}_{A,k}^{-\mu \alpha}{\left[{f}_{O}\left({D}_{ik}\right){f}_{S,m}\left({D}_{ik} \right)\right]}^{\alpha}},$$

where the variables in residential location $i$ are offset. This is the standard logit form obtained from the discrete choice model.

The objective and subjective trip cost functions from locations $i$ to $j$ are formulated as a monotonic function of trip distance as follows:

7 $${f}_{O} \left({D}_{ij}\right)={D}_{ij}^{\delta} \quad {\mathrm{and}} \quad {f}_{S,m}\left({D}_{ij}\right)={D}_{ij}^{{\beta}_{jm}},$$

where $\delta \le 0$ and ${\beta }_{jm}\le 0$ are the distance decay parameters for objective and subjective trip costs, respectively. When ${\beta }_{jm}=0$ and ${f}_{S,m}\left({D}_{ij}\right)=1$, this is a standard homogeneous assumption in distance decay parameter in the literature of migration and commuting choice. Martínez and Veigas (2013) examine several functional forms of distance decay function^41^. Halás et al. (2014) further discuss distance decay functions for daily travel-to-work^42^.

By inserting Eqs. (7) into Eq. (6), the trip probability from locations $i$ to $j$ for the purpose $m$ can be expressed as follows:

8 $${\pi}_{ijm} = \dfrac{{B}_{jm}{P}_{A,j}^{-\mu\alpha}{D}_{ij}^{\alpha \left(\delta+{\beta}_{jm}\right)}}{{\sum}_{k=1}^{N}{B}_{km}{P}_{A,k}^{-\mu \alpha}{D}_{ik}^{\alpha\left(\delta+{\beta}_{jm}\right)}}$$

Note that the distance decay parameter consists of three parameters: the perceived attractiveness of the trip destination $({\beta }_{jm}$), the trip costs proportional to trip distance ($\delta$), and the shape parameter ($\alpha$). The heterogeneity of the distance decay parameter originates only from the parameter ${\beta }_{jm}$.

Equation (8) captures the individual decision-making process for the choice of trip destination. The trip probability from locations $i$ to $j$ is likely to be high in location $j$ with lower amenity price ${P}_{A,j}$. The trip probability from locations $i$ to $j$ decreases as the trip distance increases ${D}_{ij}$. The trip probability is higher in the trip destinations with higher attractiveness that lowers subjective distance decay parameter ${\beta }_{jm}$. This study aims to recover spatial and temporal variations in the distance decay parameter included in Eq. (8) as a regional attractiveness.

### Estimation procedure of regional attractiveness index

Although the trip probability of each individual cannot be directly observed, the realized trip flows are observable. This study estimates the distance decay parameter by fitting the mobility data to the model. The expected number of trip flows ${T}_{ijm}\ge 0$ is expressed as ${\pi }_{ijm}{L}_{i}$, where ${L}_{i}$ is the total population in location $i$. Taking logarithms on both sides, the gravity equation for trip flows can be expressed as follows:

9 $${T}_{ijm}={\mathrm{exp}}\left({\phi}_{jm}{\mathrm{log}}{D}_{ij}+{\kappa}_{jm}+{\psi}_{im}\right),$$

where

10 $${\phi}_{jm} = \alpha \left(\delta+{\beta}_{jm}\right),\; {\kappa}_{jm}=-\mu \alpha \,{\mathrm{log}}{P}_{A,j}+{B}_{jm}, \; {\psi}_{im} = {\mathrm{log}}{L}_{i}-{\mathrm{log}}\left({\sum}_{k=1}^{N}{B}_{km}{P}_{A,j}^{-{\mu}{\alpha}}{D}_{ik}^{{\alpha}({\delta}+{\beta}_{km})}\right).$$

The regional attractiveness index proposed by this study is estimated as ${\phi }_{jm}\le 0$, which captures the extent to which trip destination locations attract people from other locations. The regional attractiveness index is expected to have negative values, and locations with values close to zero are more attractive, suggesting that these locations attract people from more distant locations with less trip costs. The unit of the regional attractiveness index is defined as a dimensionless quantity because it is estimated as an elasticity in the regression.

The key point in the gravity equation of trip flows is that the regional attractiveness index can be estimated from the observed interregional trip flows even though the trip preference of each individual is unobservable. However, the perceived attractiveness of the trip destination ${\beta }_{jm}$, the distance decay parameter $\delta$, and the shape parameter $\alpha$ cannot be distinguished separately and are estimated as a single parameter ${\phi }_{jm}$.

Silva and Tenreyro (2006) suggested a method for estimating the gravity equation^43^. See also Ramos (2016), who summarized previous studies on the gravity equation in migration^44^. Therefore, this study employs their approach of the Poisson regression to consider zero-flow issues. The destination-fixed Poisson regression model is as follows:

11 $${\mathrm{Pr}}\left({T}_{ij}={t}_{ij}\right)=\frac{{\mathrm{exp}}\left(-{{\lambda}}_{ij}\left({{\boldsymbol{\theta}}}_{jm}\right)\right){\left({\lambda}_{ij}\left({{\boldsymbol{\theta}}}_{jm}\right)\right)}^{{t}_{ij}}}{{t}_{ij}!}, \; {t}_{ij}=0,1,2,\dots, \; {\lambda}_{ij}\left({\boldsymbol{\theta}}_{jm}\right) \equiv {\mathrm{exp}}\left({\phi}_{jm}{\mathrm{log}}{D}_{ij}+{\kappa}_{jm}\right),$$

where ${t}_{ij}$ is the number of trip flows, and ${{\boldsymbol{\theta}}}_{jm}=\left({\phi }_{jm}, {\kappa }_{jm}\right)$ is the parameter vector, including the constant term ${\kappa }_{jm}$ and the parameter of the regional attractiveness index ${\phi }_{jm}$.

Note that Poisson regression of Eq. (11) is estimated by fixing the destination location, which leads to a heterogeneous distance decay parameter for the destination location $j$. Therefore, the fixed effect ${\psi }_{i}$ in Eq. (9) is omitted in Eq. (11). We do not further include control variables, such as population, in the origin location $i$ in Eq. (11) to capture the aggregate attractiveness of trip destinations by the coefficient parameter ${\phi }_{jm}$. If the number of observations (i.e., the number of positive trip flows) is less than 10, the distance decay parameter is not estimated.

This study incorporates an additional condition when estimating the Poisson regression of Eq. (11) to mitigate potential biases resulting from neighboring outliers. The spatial distribution of municipal areas varies, with some municipalities exhibiting relatively small areas, while others demonstrate expansive dimensions. This variation in municipal area suggests that cross-border travel may be relatively straightforward for smaller municipalities. Individuals residing in proximity to the border are also likely to influence the frequency of cross-border travel. This statistical issue arising in the spatial analysis is referred to as a modifiable area unit problem (MAUP). MAUP is defined as a challenge in analyzing spatial data that arises from variations in units or areas defined for data collection or analysis.

To control for overly close connections between neighboring municipalities with outliers, this study considers the diameter of the municipal area as an intra-municipal distance. In the event that inter-municipal trip flows fall below the specified diameter of the municipal area, they are classified as internal trip flows and are excluded from the sample. The diameter of each municipality (km) is calculated as $2\sqrt{{\mathrm{Area}}_{j}/\pi }$, where ${\mathrm{Area}}_{j}$ is the area of municipality $j$ and $\pi$ is the circular ratio.

Regarding the Person Trip Survey, this study ran 3,024 ($=432\times 7$) regressions of the gravity equation for each destination location $j$ for each trip purpose $m$. If the number of observations (i.e., the number of positive trip flows) is less than 10, the distance decay parameter was not estimated to avoid the small sample bias.

Regarding the mobile phone data, this study runs 1,896 regressions for each category to estimate the regional attractiveness index because there are 1,896 municipalities and wards in Japan, including the 23 wards of Tokyo and wards of cities designated by government ordinance. Thus, in total, this study ran 546,048 ($=\mathrm{1,896}\times 12\times 2\times 3\times 4$) regressions of the gravity equation for each destination location $j$ for 12 months, day type (weekday and weekend/holiday), gender (total, male, and female), and age group (total, 15–39, 40–59, and 60 and above).

One may consider whether the geographical unit leads to a statistical bias; however, the municipal unit is considered sufficiently small to render this concern moot. Japan is comprised of 1,896 municipalities and wards, including the 23 wards of Tokyo and the wards of cities designated by government ordinance. For the sake of comparison, the United States is comprised of 3,244 counties and county equivalents, while the European Union is comprised of 1,165 regions at the NUTS 3 level.

### Intuitive interpretation of regional attractiveness index

The regional attractiveness index proposed in this study takes values of zero or less, with values closer to zero indicating greater regional attractiveness. In this context, “regional attractiveness” refers to a region’s attractiveness as a trip destination, measured by its ability to attract people from distant regions. It is important to note that this index does not include intra-municipal mobility or data on people flowing from nearby municipalities. Additionally, it does not represent the attractiveness of a region as a place to live.

**Fig. 11:**
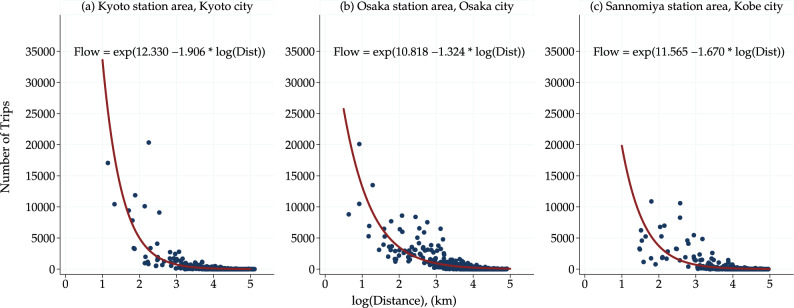
Scatter plots of the number of total trips and bilateral distance from person trip survey. Trip flows within the diameter of each area are excluded as internal trip flows. The zone codes of Kyoto, Osaka, and Sannomiya station areas, defined in the Person Trip Survey, are 31,230, 51,110, and 71,230, respectively.

**Fig. 12:**
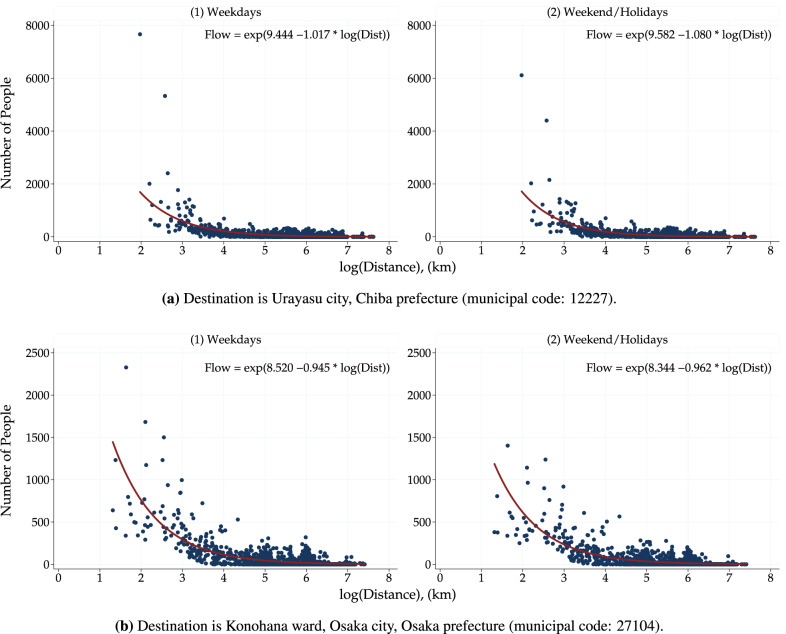
Scatter plots of the number of people and bilateral distance from mobile phone data. Trip flows within the diameter of each municipality are excluded as internal trip flows. Total flows for all people in August 2016 are visualized.

To intuitively understand the regional attractiveness index, a scatter plot of the number of people traveling between two locations and the distance traveled (logarithmic values) is useful. Figure 11 and Fig. 12, based on the Person Trip Survey and the Mobile Spatial Statistics respectively, demonstrate a negative correlation between trip frequency and distance. The number of trips was less likely to involve long-distance mobility. The fitted curves gradually decrease with the distance, and the slope of these curves represents the regional attractiveness index. A gentle slope that decreases gradually over a long distance indicates a high regional attractiveness index. Conversely, if the slope is steep and decreases rapidly, the regional attractiveness index becomes low. More precisely, the estimated coefficient parameter of the logarithm of the travel distance in the equations shown in Fig. 11 and Fig. 12 coincides with the regional attractiveness index. The regional attractiveness index is $-1.906$ in in Fig. 11(a), $-1.324$ in Fig. 11(b), and $-1.670$ in Fig. 11(c), respectively. In Fig. 12(a), the regional attractiveness index is $-1.017$ on weekday and $-1.080$ on weekend/holiday. In Fig. 12(b), the regional attractiveness index is $-0.945$ on weekday and $-0.962$ on weekend/holiday. Note that since the number of trips includes zero, a nonlinear regression model that takes zero into account (here, Poisson regression) is used instead of a log–log linear regression model.

The proximity of this coefficient parameter to zero (resulting in a flatter curve) suggests that even individuals residing at greater distances may be strongly attracted. Our observations indicate that the influence of distance friction is substantial, impeding the ability to travel over greater distances. Generally, travel costs increase as the distance traveled increases, but attractive areas have factors that reduce these subjective travel costs.

The advantage of this regional attractiveness index lies in its capacity to facilitate meaningful comparisons across regions and over time, provided that the same human mobility data and model are employed for estimation. For instance, it is possible to determine the position of one’s own municipality within the context of all municipalities as a ranking. Furthermore, when an event is hosted in a specific region, it is probable that a considerable number of individuals will be present, enabling the assessment of the extent to which the regional attractiveness index has been elevated.

A notable benefit of the proposed method is its independence from regional size, a characteristic that distinguishes it from alternative approaches. Conventional attractiveness metrics, reliant upon zone-specific constants, are contingent upon regional characteristics, including population size and economic activity levels^18,21,22^. However, the proposed index is designed to ensure equitable comparability between large and small regions.

## Supplementary Information

Supplementary Information.

## Data Availability

Please read the instructions on my GitHub (https://github.com/keisukekondokk/measuring-attractiveness-trip-destinations). The original data used in this study are publicly available, but restrictions apply to redistributing a part of the data so as not to infringe on third parties’ rights. The Stata code is available on my GitHub: (https://github.com/keisukekondokk/measuring-attractiveness-trip-destinations). The R code for the web application is available on my GitHub: (https://github.com/keisukekondokk/regional-attractiveness-kansai) and (https://github.com/keisukekondokk/regional-attractiveness-japan-en).
