# Supplementary material for: Measuring the attractiveness of trip destinations based on human mobility data
Source: Sci Rep. 2025 Nov 27;15:45485. doi: 10.1038/s41598-025-29023-0 (PMC12748571; doi:10.1038/s41598-025-29023-0)
Supplement: Supplementary file 1 — Supplementary Information. [file 41598_2025_29023_MOESM1_ESM.pdf]

Supplementary Material
Measuring the attractiveness of trip destinations based on human flow data

Keisuke Kondo[1, 2, *]

1. Research Institute of Economy, Trade and Industry
2. Research Institute for Economics and Business Administration, Kobe University

Appendix A. Web Application

This study presents two web applications that visualize estimated regional attractiveness indices.

Figure S.1 is a screenshot of the regional attractiveness index visualization system estimated from the Person Trip Survey in Japan's Kansai region.

Figure S.2 is a screenshot of the visualization system for the regional attractiveness index estimated from human mobility data based on mobile phones throughout Japan.

[Figures S.1–S.2]

* Corresponding Author. Research Institute of Economy, Trade and Industry. 1-3-1 Kasumigaseki, Chiyoda-ku, Tokyo, 100-8901, Japan. (e-mail: kondo-keisuke@rieti.go.jp).

The views expressed in this study are exclusively those of the author and do not represent those of the author's affiliated organizations, the Research Institute of Economy, Trade and Industry (RIETI) and the Research Institute for Economics and Business Administration (RIEB), Kobe University.

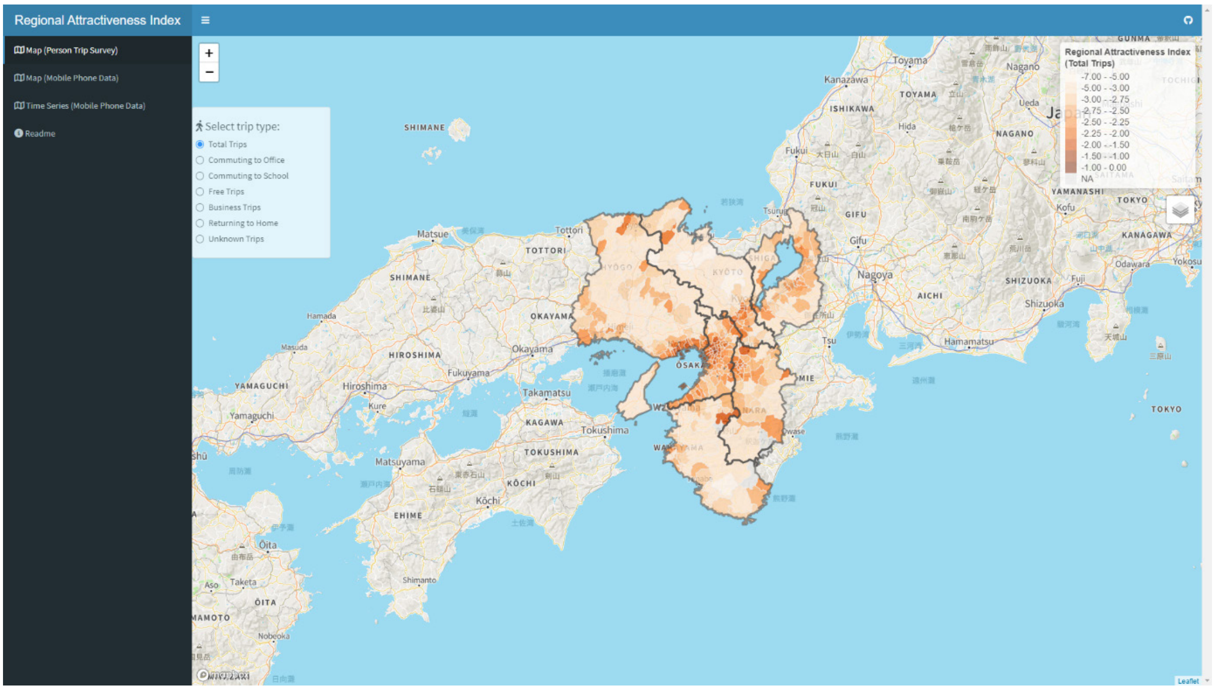

Figure S.1. Visualizing regional attractiveness index estimated from Person Trip Survey in the Kansai area of Japan.

URL: https://keisuke-kondo.shinyapps.io/regional-attractiveness-kansai/

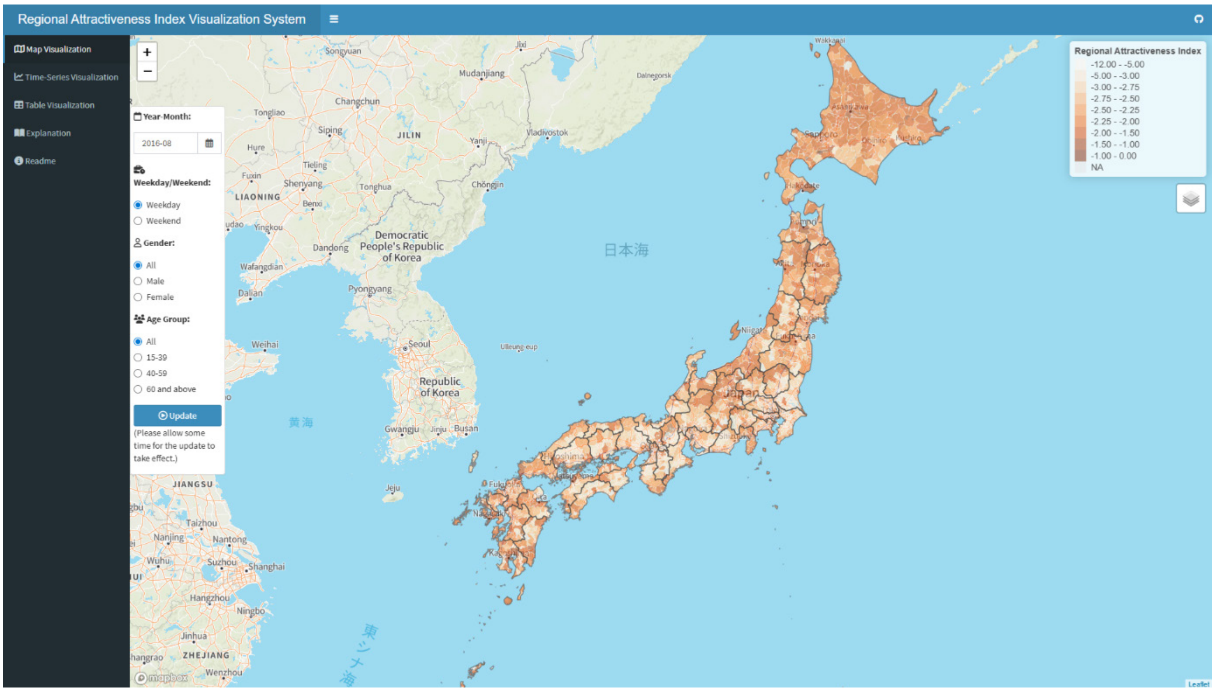

Figure S.2. Visualizing regional attractiveness index estimated from the human mobility data based on mobile phones throughout Japan.

URL: https://keisuke-kondo.shinyapps.io/regional-attractiveness-japan-en/

Appendix B. Hot and Cold Spot Analysis

The standard method for hot and cold spot analyses in spatial statistics is the Getis–Ord $G_i^*(d)$ statistic, which tests whether a region and its neighboring regions form a spatial cluster. The Stata's getisord command is used to calculate the Getis–Ord $G_i^*(d)$ statistic[1].

Getis and Ord proposed the following statistic[2,3]:

$$G_i^*(d) = \frac{\sum_{k=1}^{N} w_{ik}(d) x_k}{\sum_{k=1}^{N} x_k}, \tag{S.1}$$

where $w_{ik}(d)$ denotes the ijth element of the spatial weight matrix. Each element takes a binary value, as follows:

$$w_{ik}(d) = \begin{cases} 1, & \text{if } d_{ij} < d \text{ for all } i, j \\ 0, & \text{otherwise,} \end{cases} \tag{S.2}$$

where d is the threshold distance. In this study, the threshold distance d is set to 10 km.

The numerator of the Getis–Ord $G_i^*(d)$ statistic represents the local sum of variable x within a circle of radius d km, and the denominator represents the total sum of variable x. Therefore, those regions with higher (lower) shares of variable x are detected as hot (cold) spots. The null hypothesis is complete spatial randomness, and rejection of the null hypothesis indicates an outlier in the geographical space.

The standardized $G_i^*(d)$ can be viewed as z value of Getis–Ord $G_i^*(d)$, as follows:

$$\text{Standardized } G_i^*(d) = \frac{G_i^*(d) - \mathrm{E}[G_i^*(d)]}{\sqrt{\mathrm{Var}[G_i^*(d)]}}, \tag{S.3}$$

where $\mathrm{E}[G_i^*(d)]$ and $\mathrm{Var}[G_i^*(d)]$ represent the expected values and variance of $G_i^*(d)$ under the null hypothesis, respectively. The distribution of the standardized $G_i^*(d)$ approaches a standard normal distribution as N approaches infinity. When the standardized $G_i^*(d)$ takes a positive (negative) value and falls within the critical region, region i is identified as a hot (cold) spot. The critical values for hot and cold spots are approximately ± 1.96 and ± 2.58 at the 5% and 1% significance levels, respectively.

References

1. Kondo, K. Hot and Cold Spot Analysis Using Stata. *The Stata Journal* **16**, 613–631; 10.1177/1536867X1601600304 (2016).

2. Getis, A. & Ord, J. K. The analysis of spatial association by use of distance statistics. *Geographical Analysis* **24**, 189–206; 10.1111/j.1538-4632.1992.tb00261.x (1992).

3. Ord, J. K. & Getis, A. Local spatial autocorrelation statistics: Distributional issues and an application. *Geographical Analysis* **27**, 286–306; 10.1111/j.1538-4632.1995.tb00912.x (1995).
